# Endoscopic-Assisted evacuation vs. burr-hole drainage for chronic subdural hematoma: a retrospective comparative study

**DOI:** 10.3389/fsurg.2026.1759497

**Published:** 2026-05-12

**Authors:** Yang Mi, Chunhong Wang, Xiaohui Yao, Chunlei Ju, Kai Yang, Xulei Hu, Hao Li, Haiyang Su, Hongming Ji

**Affiliations:** 1 The Neurosurgery Department of Shanxi Provincial People’s Hospital, Shanxi Medical University, Taiyuan, China; 2 The Neurosurgery Department of Shanxi Provincial People’s Hospital, Taiyuan, China; 3 The Neurosurgery Department of Quwo People’s Hospital, Linfen, China

**Keywords:** burr-hole drainage, chronic subdural hematoma, direct visualization, endoscopic, real-timehemostasis

## Abstract

**Objective:**

This study aimed to compare the perioperative outcomes of endoscopic-assisted evacuation vs. burr-hole drainage methods in the chronic subdural hematoma (CSDH).

**Methods:**

This retrospective cohort study included consecutive surgical cases of CSDH treated at Shanxi Provincial People's Hospital. After eligibility screening, 40 patients who underwent endoscopic-assisted evacuation and 158 who underwent burr-hole drainage were included in the analysis. Postoperative outcomes were systematically evaluated across intraoperative parameters, clinical and laboratory measures, procedural costs, and length of hospital stay.

**Results:**

Patients who underwent endoscopic-assisted evacuation had significantly lower residual hematoma rates compared to those who underwent burr-hole drainage (35.00% vs. 54.78%, *p* = 0.0255). In the multiple regression analysis, the endoscopic group demonstrated improved neurological outcomes compared to the burr-hole group, with an odds ratio of 0.30 (95% CI: 0.12–0.63; *p* = 0.0021) for achieving a good functional outcome (lower mRS). However, endoscopic-assisted evacuation was associated with longer operative time, averaging 40.12 min longer (*p* < 0.0001) and higher hospitalization costs, averaging ¥9,600 more (*p* < 0.0001). Occurrence of postoperative complications such as intracranial pneumocephalus and hematoma recurrence were not significantly different between the two groups. Hemoglobin count was lower in the endoscopy group than in the burr-hole group (127.90 ± 14.93 vs. 133.00 ± 14.35 g/L, *p* = 0.0514) although no anemia-related complications occurred in the endoscopic group.

**Conclusion:**

Endoscope-assisted evacuation enables more thorough clearance of hematoma and leads to better recovery for patients, without increasing procedural trauma or postoperative complications, though its higher cost may limit accessibility. These findings may help inform surgical decision-making and resource allocation.

## 1 Introduction

Chronic subdural hematoma (CSDH) (1) is a common neurosurgical condition, pathologically characterized by abnormal accumulation of blood, cerebrospinal fluid, and fibrin degradation products in the subdural space, often accompanied by hematoma capsule formation and septate structures (2, 3). CSDH can cause brain compression, leading to progressive neurological deterioration and even death, so emergency surgery is necessary under specific circumstances (4). Epidemiological data indicate an annual incidence of up to 58.1 per 100,000 in individuals over 65 years old (5), and the postoperative recurrence rate in elderly patients is 3–5 times higher than those who are younger (6). With the accelerated aging of the population, the incidence of CSDH is rising significantly. Researchers estimate that by the year 2030, the incidence of CSDH will exceed the incidence of brain tumors and become the most common craniosurgical disease (6, 7).

Diagnosis of CSDH primarily relies on imaging evaluation. Computed tomography (CT) scans can rapidly identify isodense or hypodense crescent-shaped lesions with high sensitivity for acute hemorrhage, while magnetic resonance imaging (MRI) particularly the fluid-attenuated inversion recovery sequence clearly displays multiloculated septations (8), the inner hematoma membrane, and fibrotic features, providing critical value for the diagnosis of complex CSDH (9). The recurrence mechanism involves multiple pathological factors, including drainage obstruction due to thickening of the hematoma capsule, inflammatory responses triggered by residual blood clots, age-related brain atrophy, and coagulation abnormalities (10, 11). Notably, many patients have a history of anticoagulant use, which further increases the risk of postoperative rebleeding (12).

Currently, the primary surgical interventions for CSDH are burr-hole drainage, middle meningeal artery embolization (MMAE) and endoscope-assisted hematoma evacuation (13, 14). MMAE has been proposed as an additional treatment to mitigate recurrence risk in CSDH, with growing evidence supporting its utility particularly in elderly patients receiving anti-platelet, anticoagulant medications, or with coagulopathies. It may be employed both as a surgical adjunct and as an independent nonsurgical intervention (6). As the traditional standard procedure, burr-hole drainage offers advantages of operational simplicity and minimal invasiveness (15). However, its limitations are significant: constrained by the narrow visual field through the small bone window, it often fails to thoroughly evacuate fibrin degradation products within fibrous septa and postoperative pneumocephalus, leading to potential hematoma retention (16, 17). The intraoperative lack of real-time hemostatic capability may result in missed minor bleeding points. Furthermore, the procedure carries a high incidence of postoperative intracranial air accumulation, and issues such as erroneous placement of the drainage tube into the brain parenchyma or insufficient depth within the hematoma cavity can further compromise therapeutic efficacy. Studies indicate postoperative hematoma recurrence rate can reach 10%–20% (18, 19) after burr-hole drainage, with particularly poorer prognoses in elderly patients and those with coagulation disorders.

In recent years, the endoscope-assisted surgery technique has been applied in the treatment of CSDH. The endoscope provides a broader inspection of subdural space and does less harm to patients. Although some studies have established that endoscopic surgery surpasses traditional techniques in the management of CSDH (14, 20–22), existing evidence is limited and primarily consists of small case series or studies lacking comprehensive comparative analyses, and some scholars express concern that its relatively greater surgical invasiveness might offset potential benefits, which has thus far prevented this technique from becoming a routine treatment of complex CSDH.

To directly assess the effectiveness and safety of endoscopic-assisted evacuation compared with conventional burr-hole drainage, we conducted a single-center retrospective analysis of 198 patients with chronic subdural hematoma (CSDH) who underwent either procedure. Clinical, imaging, and laboratory data were systematically collected and analyzed. Through comprehensive comparison of outcomes between the two surgical approaches, this study aimed to identify the optimal surgical management strategy for patients with CSDH.

## 2 Materials and methods

### 2.1 Analysis cohort selection

This single-center retrospective cohort study, conducted in accordance with the STROBE statement, included consecutive patients diagnosed with CSDH in the Department of Neurosurgery at Shanxi Provincial People's Hospital between March 2023 and February 2025. Inclusion criteria were: ① age ≥ 55 years; ② confirmed diagnosis of CSDH (hematoma thickness ≥ 10 mm) by cranial CT scan or MRI; and ③ undergoing surgical treatment under general anesthesia (either unilateral burr-hole drainage or endoscope-assisted evacuation). Data was collected via the electronic medical record system using standardized forms, covering baseline characteristics, surgical parameters, perioperative indicators, and relevant laboratory tests pre- and post-operation. Outcome data were independently assessed by two blinded neurosurgeons, with discrepancies resolved through discussion between the two surgeons. The study received approval from the Ethics Committee of Shanxi Provincial People's Hospital. As a retrospective analysis, patient informed consent was waived, and data storage complied with the Personal Information Protection Law.

### 2.2 Surgical procedure

All patients underwent surgery under general anesthesia in the supine position. The surgical incision was made at the thickest part of the hematoma. For patients undergoing burr-hole drainage, a straight incision approximately 3cm in length was made. A burr-hole with a diameter of about 1cm was created, and after incising the dura mater, the hematoma cavity was irrigated with normal saline via a drainage tube. For patients underwent endoscopic-assisted evacuation surgery, an incision approximately 5cm in length was made, and a small bone flap with a diameter of about 3–5cm was removed using a milling cutter. Under direct endoscopic visualization, blood clots were evacuated and hemostasis was achieved, followed by irrigation of the hematoma cavity with normal saline via the drainage tube. The procedure was concluded in all patients once the effluent ran clear with no flocculent material. All patients underwent CT scanning on the first postoperative day, and the residual hematoma and intracranial pneumocephalus were evaluated by two neurosurgeons through imaging studies. Patients were additionally administered atorvastatin calcium tablets to reduce the risk of hematoma recurrence following burr-hole or endoscopic-assisted evacuation surgery.

### 2.3 Clinical outcomes

The assessment outcomes include a comprehensive postoperative evaluation system, encompassing various factors: postoperative residual hematoma, pneumocephalus, hypoproteinemia, electrolyte disturbances, anemia, thrombus, pneumonia, subdural effusion, intracranial infection, hematoma, three-month recurrence rates, cost, length of hospital stay, operation time, drainage volume, drainage time, Glasgow Coma Scale (GCS) score, Markwalder score, modified Rankin scale (mRS) score, temperature, blood pressure, and postoperative laboratory indicators.

### 2.4 Statistical analysis

This retrospective observational study included 40 patients who underwent endoscopic-assisted evacuation and 158 patients who received burr-hole drainage. Data sets were examined for normality and equal variance using Shapiro–Wilk and Levene tests respectively before statistical analysis. Continuous variables were summarized as mean ± standard deviation (SD) if normally distributed and as median (Q1-Q3) if non-normally distributed. Between-group comparisons were performed using the independent-samples t-test for normally distributed variables and the Wilcoxon rank-sum test for non-normally distributed variables. Categorical variables were expressed as frequencies and percentages and compared using the chi-square test or Fisher's exact test, as appropriate based on expected cell counts.

To adjust for potential confounding, multiple regression analyses were performed, adjusting for five variables that showed between-group difference at baseline: Preoperative treatment, hemostatic drug usage, D-dimer level, smoking, and intracranial pressure. General linear regression models were applied for normally distributed continuous outcomes, while 50th percentile (median) quantile regression models were used for non-normally distributed outcomes. Logistic regression models were used for categorical outcomes, and Poisson regression models were applied for count or rare event outcomes. All statistical analyses were performed using SPSS Statistics software (IBM Corporation, NY, USA). A two-sided *p*-value < 0.05 was considered statistically significant.

## 3 Results

### 3.1 Analysis cohort

**Figure 1:**
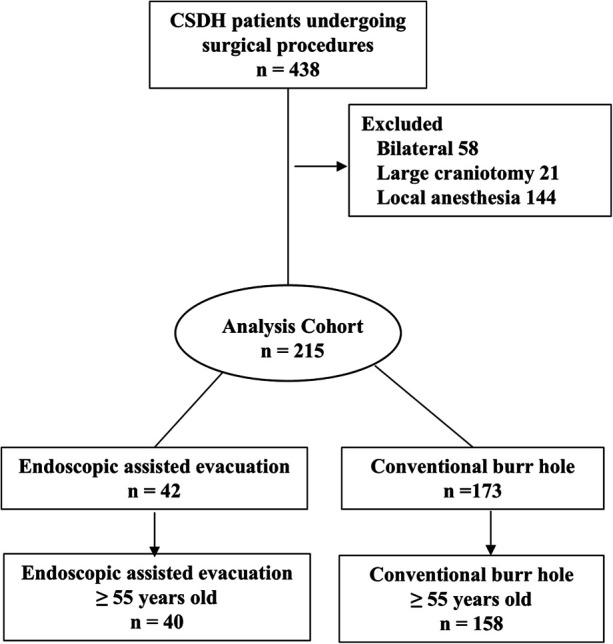
Study flow diagram.

This single-center retrospective cohort study, approved by the Ethics Committee of Shanxi Provincial People's Hospital, screened 438 patients with CSDH who underwent surgical procedures between March 2023 and February 2025. Of the 438 patients, 223 were excluded from the analysis (58 cases of bilateral hematoma evacuation, 21 cases of large craniotomy, and 144 cases of hematoma clearance under local anesthesia). Among the remaining 215 eligible patients, 42 underwent endoscope-assisted mini-craniotomy (2 patients aged < 55 years), and 173 underwent unilateral burr-hole drainage (15 patients aged < 55 years). Based on predetermined exclusion criteria (age < 55 years), 198 surgical patients (40 in the endoscopic group and 158 in the burr-hole group) were ultimately included for statistical analysis (Figure 1).

### 3.2 Demographics and preoperative characteristics

The current study included a total of 198 patients with chronic subdural hematoma, comprising an endoscopic group (*n* = 40) and a burr-hole group (*n* = 158) (Table 1). Apart from a history of preoperative treatment (*p* = 0.0222) and D-dimer levels (*p* < 0.0001), which showed statistically significant differences, no significant differences were observed in patients' demographic, radiological characteristics, clinical features, neurological assessments, comorbidities and risk factors between the two groups (Table 1). The mean age was 69 years in the endoscopic group and 70 years in the burr-hole group (*p* = 0.7686), with male patients accounting for 82.50% and 84.18%, respectively. No statistically significant differences were found in smoking, alcohol consumption, diabetes, hypertension, hematoma side, or relevant medication use. Symptoms suggesting increased intracranial pressure such as headache, dizziness, nausea and vomiting were present in 50.00% of the endoscopic group and 63.92% of the burr-hole group (*p* = 0.1066), while localizing symptoms (such as limb hemiplegia), GCS scores and mRS at admission were comparable between groups (all *p* > 0.37). The mean hematoma thickness on imaging was 23.89 ± 4.30 mm in the endoscopic group and 24.69 ± 5.79 mm in the burr-hole group (*p* = 0.3701). No significant differences were detected in patients' preoperative vital signs and laboratory measurements (complete blood count, electrolytes, liver and kidney functions) (Supplementary Table S1).

**Table 1:** Patients characteristics and preoperative assessments.

Characteristics	Burr-Hole Group *N* = 158	Endoscopic Group *N* = 40	Total *N* = 198	*P*-Values
Age, Mean ± SD, years	69.65 ± 8.54	70.10 ± 8.82	69.74 ± 8.57	0.7686
Male, *n* (%)	133 (84.18)	33 (82.50)	166 (83.84)	0.7969
				
Hematoma Thickness（mm）, Mean ± SD	24.69 ± 5.79	23.89 ± 4.30	24.54 ± 5.54	0.3701
Side (Left), *n* (%)	100 (63.29)	25 (62.50)	125 (63.13)	0.9262
Intracranial Pressure, *n* (%)	101 (63.92)	20 (50.00)	121 (61.11)	0.1066
Local Symptom, *n* (%)	107 (67.72)	30 (75.00)	137 (69.19)	0.3731
D-dimer （ng/mL), Median (Q1-Q3)	108.0 (1.3–204.0)	209.0 (117.0–378.0)	123.5 (46.5–234.5)	<0.0001
				
GCS Score, Median (Q1-Q3)	15.0 (14.0–15.0)	15.0 (14.0–15.0)	15.0 (14.0–15.0)	0.9083
Markwalder Score, Median (Q1-Q3)	2.0 (2.0–2.0)	2.0 (2.0–2.0)	2.0 (2.0–2.0)	0.7714
mRS Score, Median (Q1-Q3)	3.0 (3.0–4.0)	3.5 (3.0–4.0)	3.0 (3.0–4.0)	0.4218
				
Preoperative Treatment, *n* (%)	23 (14.56)	12 (30.00)	35 (17.68)	0.0222
Received Atorvastatin, *n* (%)	137 (86.71)	34 (85.00)	171 (86.36)	0.7785
Received Anticoagulant, *n* (%)	22 (13.92)	6 (15.00)	28 (14.14)	0.8615
Received Prophylactic AEDs, *n* (%)	144 (91.14)	36 (90.00)	180 (90.09)	0.8228
Hemostatic drug usage, *n* (%)	96 (61.15)	18 (45.00)	114 (57.87)	0.0649
				
Diabetes, *n* (%)	24 (15.19)	5 (12.50)	29 (14.65)	0.8052
Hypertension, *n* (%)	51 (32.28)	17 (42.50)	68 (34.34)	0.2239
Smoking, *n* (%)	78 (50.00)	14 (35.00)	92 (46.94)	0.0899
Drinking, *n* (%)	58 (37.18)	11 (27.50)	69 (35.20)	0.2528
BMI, Mean ± SD	24.40 ± 3.92	22.91 ± 3.22	24.10 ± 3.82	0.1011

### 3.3 Clinical and economic outcomes

We first compared clinical and economic outcomes between the burr-hole and endoscopic groups directly using chi-square or Fisher's exact tests for categorical variables and independent t-tests or Wilcoxon rank-sum tests for continuous variables. We then compared the outcomes between the two groups after adjusting for potential confounders by performing multiple regression analyses.

The endoscopic group showed a significantly lower postoperative residual hematoma rate compared to the burr-hole group (35.00% vs. 54.78%, *p* = 0.0255), while the incidence of postoperative intracranial pneumocephalus and three-month recurrence rates were comparable between groups (Table 2). Regarding operative metrics, the endoscopic group had a significantly longer operation duration (116.70 ± 54.19 vs. 74.42 ± 25.99 min, *p* < 0.001), while drainage volume and duration of subdural drainage did not differ significantly between groups (Table 2).

**Table 2:** Postoperative assessments after burr-hole and endoscopic-assisted evacuation procedures.

Outcomes	Burr-Hole Group *N* = 158	Endoscopic Group *N* = 40	Total *N* = 198	*P*-Values
Residual Hematoma, *n* (%)	86 (54.78)	14 (35.00)	100 (50.76)	0.0255
Pneumocephalus, *n* (%)	121 (77.07)	29 (72.50)	150 (76.14)	0.5449
Postoperative Hypoproteinemia, *n* (%)	56 (35.44)	13 (32.50)	69 (34.84)	0.7271
Postoperative Electrolyte Disturbance, *n* (%)	18 (11.39)	5 (12.50)	23 (11.62)	0.7876
Postoperative Anemia, *n* (%)	0 (0.00)	1 (2.50)	1 (0.51)	0.2020
Postoperative Thrombus, *n* (%)	10 (6.33)	5 (12.50)	15 (7.52)	0.1907
Postoperative Pneumonia, *n* (%)	30 (18.99)	9 (22.50)	39 (19.70)	0.6178
Postoperative Subdural Effusion, *n* (%)	5 (3.16)	1 (2.50)	6 (3.03)	1.0000
Postoperative Intracranial Infection, *n* (%)	2 (1.27)	1 (2.50)	3 (1.52)	0.4958
Postoperative Hematoma, *n* (%)	1 (0.63)	0 (0.00)	1 (0.51)	1.0000
Recurrence rate at three-month, *n* (%)	1 (0.63)	2 (5.00)	3 (1.52)	0.1045
				
Cost (x1000 Yuan), Median (Q1-Q3)	27.8 (23.3–34.0)	37.4 (29.8–41.4)	28.9 (24.4–37.3)	<0.0001
Hospital Stay (days), Median (Q1-Q3)	9.0 (7.0–12.0)	9.0 (7.0–11.0)	9.0 (7.0–11.0)	0.9369
Operation Duration (Minutes), Mean ± SD	74.42 ± 25.99	116.70 ± 54.19	83.05 ± 37.63	<0.001
Drainage Volume（mL）, (Q1-Q3)	85.0 (50.0–160.0)	95.0 (55.0–150.0)	85.0 (50.0–160.0)	0.5224
Duration of Subdural Drainage (days), Median (Q1-Q3)	2.0 (2.0–3.0)	3.0 (2.0–3.0)	2.0 (2.0–3.0)	0.1445
				
White Blood Cells(10^9/L), Mean ± SD	9.78 ± 3.01	8.83 ± 2.15	9.59 ± 2.88	0.0623
Neutrophils(10^9/L), Mean ± SD	8.48 ± 5.99	7.26 ± 2.19	8.23 ± 5.45	0.0422
Monocytes(10^9/L), Median (Q1-Q3)	0.5 (0.4–0.7)	0.5 (0.4–0.6)	0.5 (0.4–0.7)	0.1931
Lymphocytes(10^9/L), Mean ± SD	1.09 ± 0.50	0.94 ± 0.35	1.06 ± 0.47	0.0227
Platelets(10^9/L), Mean ± SD	213.90 ± 81.00	200.00 ± 75.84	211.04 ± 79.97	0.3289
Hemoglobin(g/L), Mean ± SD	133.00 ± 14.35	127.90 ± 14.93	131.92 ± 14.57	0.0514
D-dimer（ng/mL), Median (Q1-Q3)	300.0 (165.0–645.0)	354.0 (243.5–878.5)	310.5 (181.0–68.5)	0.0433
				
GCS Score, Median (Q1-Q3)	15.0 (15.0–15.0)	15.0 (15.0–15.0)	15.0 (15.0 -15.0)	0.6613
Markwalder Score, Median (Q1-Q3)	1.0 (1.0–2.0)	1.0 (0.0–2.0)	1.0 (1.0–2.0)	0.6416
mRS Score, Median (Q1-Q3)	2.0 (1.0–2.0)	1.0 (1.0–2.0)	2.0 (1.0–2.0)	0.1409

Interestingly, both neutrophils and lymphocytes were significantly higher in burr-hole group compared to endoscopic group (8.48 ± 5.99 vs. 7.26 ± 2.19 10^9/L, *p* = 0.0422 for neutrophils; 1.09 ± 0.50 vs. 0.94 ± 0.35 10^9/L, *p* = 0.0227 for lymphocytes). The hemoglobin count was lower in the endoscopic group compared to the burr-hole group (127.90 ± 14.93 vs. 133.00 ± 14.35 g/L, *p* = 0.0514). The postoperative D-dimer values remained significantly higher in the endoscopic group compared to the burr-hole group (354.0 ng/mL [243.5–878.5] vs. 300.0 ng/mL [165.0–645.0], *p* = 0.0433) (Table 2). No statistically significant differences were observed in postoperative vital signs, electrolytes, liver and kidney function (Supplementary Table S2).

The endoscopic group incurred significantly higher total costs (37.4 (29.8–41.4) × 1000 yuan vs. 27.8 (23.3–34.0) × 1000 yuan, *p* < 0.0001), though hospital stay duration was comparable between groups. The operation time is also longer in the endoscopic group compared to the burr-hole group (95% CI 27.78–52.46 Minutes, *P* < 0.0001) (Table 2).

After adjusting for potential confounders, the endoscopic group showed a 55% reduction in the odds of residual hematoma compared with the burr-hole group (adjusted OR = 0.45; 95% CI 0.21–0.97; *P* = 0.0422) (Table 3). While the mRS at discharge showed no significant difference between groups (1.0 (1.0–2.0) vs. 2.0 (1.0–2.0), *p* = 0.1409) in direct comparison, the endoscopic group demonstrated significantly lower mRS scores compared to the burr-hole group, with an OR for achieving a good functional outcome of 0.30 (95% CI: 0.12–0.63; *p* = 0.0021), indicating improved outcomes after adjusting for confounding variables (Table 3). The difference of neutrophils counts between the two groups changed slightly in multiple regression analysis. The increase in neutrophils was, on average, 1.02 × 10^9^/L less (*p* = 0.0626) in the endoscopic group compared to the burr-hole group. The lymphocytes count between the two groups was no longer significantly different (*p* = 0.1596). Multiple regression analysis showed that hemoglobin decreased by an average of 5.48 g/L more in the endoscopic group (adjusted coefficient = −5.48; 95% CI, −9.10 to −1.90; *p* = 0.0032), revealing a more significant difference between the two groups after adjusting for confounders (Table 3). The cost remains significantly higher in the endoscopic group vs. the burr-hole group (95% CI 4.54–12.01 × 1000 yuan, *P* < 0.0001) (Table 3). The results of vital signs, kidney function, and liver function remained comparable between the endoscopic and burr-hole groups in multiple regression analysis (Table 3).

**Table 3:** Multiple regression comparison of clinical outcomes, laboratory measures and vital signs after burr-hole drainage and endoscopic-assisted evacuation.

Outcomes	Treatment Effect	95% C.I.	Statistics	*P*-Values
Cost (x1000 Yuan), Coefficient^a^	8.23	4.54–12.01	4.3	<0.0001
Hospital of Stay (Days), Coefficient^a^	−1.01	−2.43–0.41	−1.40	0.1632
Operation time (Minutes), Coefficient^b^	40.12	27.78–52.46	6.41	<0.0001
Drainage Time (days), Coefficient^a^	0.00	−0.34–0.34	0.00	1.0000
Drainage Volume(mL), Coefficient^a^	4.69	−27.36–36.74	0.29	0.7730
				
Residual Hematoma, OR^c^	0.45	0.21–0.97	4.13	0.0422
Pneumocephalus, OR	0.70	0.30–1.64	0.66	0.4152
Postoperative Pneumonia, OR	1.14	0.44–2.95	0.07	0.7880
Postoperative Subdural Effusion, OR	0.90	0.09–8.92	0.01	0.9257
Postoperative Hypoproteinemia, OR	0.85	0.38–3.69	0.18	0.6745
Postoperative Electrolyte Disturbance, OR	1.48	0.44–4.98	0.41	0.5239
Postoperative Thrombus, OR	1.36	0.40–4.59	0.24	0.6254
Recurrence rate at 3 months, RR^d^	11.8	0.89–132.95	3.47	0.0626
				
Temperature (℃), Coefficient^a^	0.03	−0.14–0.20	0.31	0.7582
Systolic Blood Pressure(mmHg), Coefficient^b^	−1.03	−7.71–5.66	−0.30	0.7623
Diastolic Blood Pressure(mmHg), Coefficient^b^	−0.31	−5.10–4.48	−0.13	0.8988
Mean Arterial Pressure(mmHg), Coefficient^b^	−0.55	−5.49–4.39	−0.22	0.827
				
Potassium（mmol/L), Coefficient^b^	−0.03	−0.16–0.11	−0.39	0.6998
Sodium（mmol/L), Coefficient^b^	1.18	0.03–2.35	2.02	0.0454
Chloride（mmol/L), Coefficient^b^	0.75	−0.54–2.03	1.14	0.254
White Blood Cells(10^9/L), Coefficient^b^	−1.00	−2.08–0.07	−1.84	0.067
Neutrophils(10^9/L), Coefficient^b^	−1.02	−2.09–0.05	−1.88	0.0619
Monocytes(10^9/L), Coefficient^a^	−0.08	−0.17–0.01	−1.71	0.0883
Lymphocytes(10^9/L), Coefficient^b^	−0.14	−0.32–0.05	−1.41	0.1596
Platelets(10^9/L), Coefficient^b^	−8.30	−19.00–2.37	−1.54	0.1265
Hemoglobin(g/L), Coefficient^b^	−5.48	−9.10–−1.90	−2.99	0.0032
Albumin(g/L), Coefficient^b^	−0.44	−2.31–1.44	−0.46	0.6453
Postoperative D-dimer（ng/mL), Coefficient^b^	18.28	−95.28–131.85	0.32	0.7511
Creatinine（umol/L), Coefficient^b^	1.16	−2.30–4.62	0.66	0.5082
Blood Urea Nitrogen(mmol/L), Coefficient^b^	0.16	−0.70–1.02	0.37	0.7124
Aspartate Aminotransferase(IU/L), Coefficient^a^	−0.54	−3.15–2.07	−0.41	0.6844
Alanine Aminotransferase(IU/L), Coefficient^a^	−2.00	−3.72–−0.28	−2.30	0.0229
				
GCS Score, OR	0.70	0.15–3.37	0.20	0.6558
Markwalder Score, OR	0.70	0.33–1.48	0.35	0.3474
mRS Score, OR	0.30	0.12–0.63	9.47	0.0021

a Coefficient estimated from 50 percentile multiple quantile regressions.

b Coefficient estimated from multiple linear regressions.

c OR is odds ratio from multiple Logistic regressions.

d RR is relative risk from multiple Poisson regressions.

**Figure 2:**
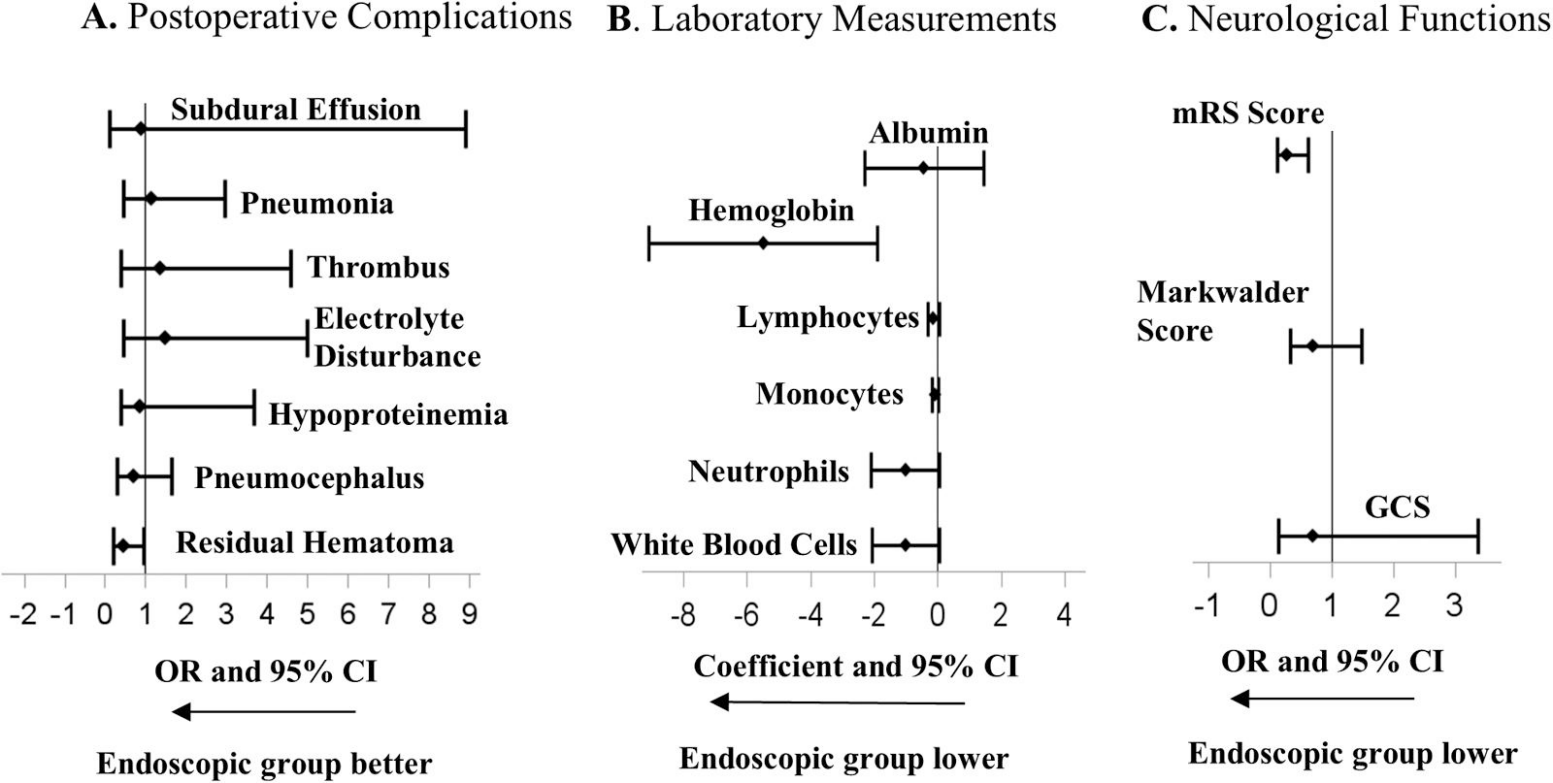
Forest plots illustrating postoperative outcome comparisons between endoscopic and burr-hole groups based on multiple regression analyses. **A**. Odds ratios (OR) and 95% confidence intervals (CI) were estimated from multiple logistic regressions for postoperative complications. **B.** Treatment coefficients and 95% CI were estimated from multiple linear regression for laboratory measurements. **C**. OR and 95% CI from multiple proportional logistic regressions for neurological function assessments. Lower mRS score corresponds to better neurological outcome.

The multiple regression analysis comparing postoperative complications, laboratory measures, and neurological functions between the two groups is summarized in a forest plot (Figure 2).

## 4 Discussion

This retrospective analysis compared the perioperative outcomes of endoscopic-assisted evacuation vs. burr-hole drainage for CSDH and found that the endoscopic group had lower postoperative residual hematoma rate and improved neurological function (lower mRS) at discharge, although it requires longer operative time and higher cost, primarily attributable to the need for more expensive equipment, neurosurgeons skilled in endoscopic techniques and the additional surgical consumables required in endoscopic-assisted hematoma evacuation. Occurrence of postoperative complications such as intracranial pneumocephalus and hematoma recurrence were not significantly different between the two groups.

**Figure 3:**
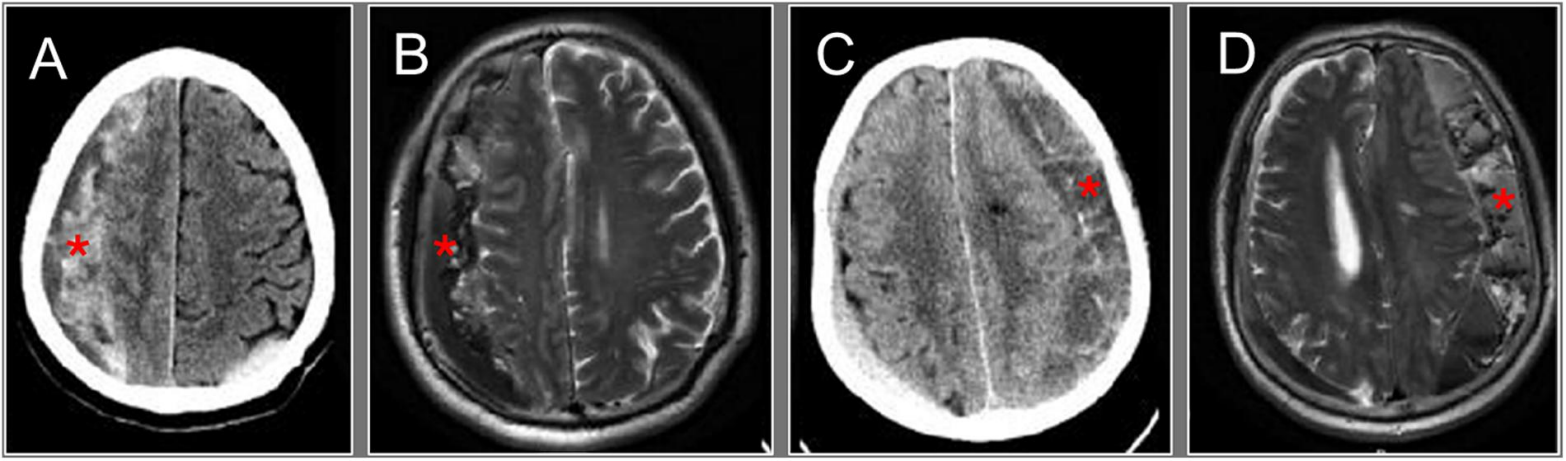
The preoperative cranial CT scan **(A** and **C)** and MRI **(B** and **D)** from two patients. Images **A, B** and **C, D** are from the same patient respectively. The asterisks in A-D indicate the locations of the subdural hematoma presented as mixed-density or heterogeneous signal intensity. This radiographic feature suggests the possible presence of fibrous membrane septa and residual blood clots within the hematoma cavity. Generally, the more heterogeneous the density of the hematoma, the more complex the structure of the intracavitary fibrous membranes tends to be. Intraoperatively, with the assistance of a endoscope, these fibrous membranes can be directly observed diffusely distributed throughout the hematoma cavity, which impedes the placement of drainage tubes and irrigation of the cavity. This constitutes a key factor making complete evacuation of the hematoma particularly challenging.

**Figure 4:**
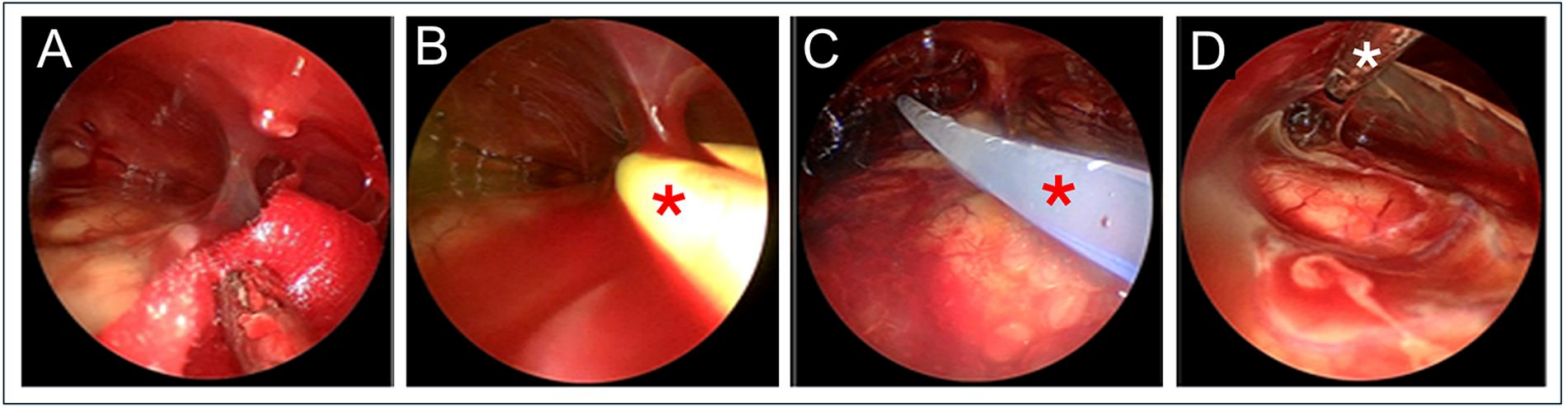
Endoscopic-assisted hematoma evacuation. Under endoscopic assistance, the hematoma cavity can be accessed, allowing for real-time observation of the distribution of fibrous membrane septa and blood clots within the cavity. **A.** Fine, dense reticular network of fibrous membranes is seen diffusely distributed within the hematoma cavity. **B**. During endoscopic assisted evacuation, small blood vessels are coagulated using bipolar electrocautery (asterisk), and fibrous membranes are removed. **C.** The drainage tube (asterisk) is safely placed into the hematoma cavity, and blood clots within the cavity are irrigated. **D**. The surgeon manipulates the endoscope with one hand to observe the hematoma cavity while using the other hand to operate the suction device (asterisk), clearing residual blood clots from the surface of the brain tissue.

Endoscopic-assisted evacuation has been shown to be more effective than burr-hole drainage for complete hematoma removal, particularly those with complex structures (23). The presence of mixed-density hematoma on cranial CT/MRI (Figure 3) indicates the existence of fibrous membrane septations within the hematoma cavity (24). A greater number of these membranes correspond to increased structural complexity which has been visually confirmed under direct intraoperative endoscopic observation (Figure 4). Irregular fibrous membranes are often diffusely distributed throughout the hematoma cavity in a dense, reticular pattern, forming multiple septations. These membranes contain extensive microvascular networks, which may be a primary source for postoperative hematoma recurrence (25). Under endoscopic guidance, the surgeon can hold the endoscope in one hand to explore the hematoma cavity and use instruments such as a suction device or bipolar electrocautery in the other hand to evacuate blood clots, achieve hemostasis, and lyse septations, thereby thoroughly removing intracranial clots and bleeding points (Figure 3D). The presence of numerous fibrous membrane septations can lead to incomplete irrigation of the hematoma during surgery. Residual blood clots persistently induce an inflammatory response against the brain tissue (26, 27), a finding consistent with the higher postoperative neutrophil count observed in the burr-hole group compared to the endoscopy group. These residual clots represent a major factor affecting patient prognosis, as they sustain ongoing inflammation. Several studies have suggested that inflammation-related indicators may serve as reliable markers for evaluating treatment efficacy and predicting outcomes in patients with CSDH (28, 29). Generally, surgical procedures with greater trauma lead to higher neutrophil counts; however, the observation of elevated neutrophil counts in the burr-hole drainage group suggests that residual blood clots may be a more significant driver of the patient's inflammatory response. Furthermore, relying solely on drainage tube irrigation makes it difficult to completely evacuate blood clots from the hematoma cavity. Failure to clear these clots promptly prolongs the duration of drainage tube placement, creating a favorable environment for bacterial growth and increasing the risk of intracranial infection. Thus, the ability to directly visualize and lyse fibrous septations under endoscopic guidance represents a critical advantage in achieving complete hematoma evacuation and minimizing postoperative complications. Thorough hematoma evacuation assisted by endoscopic may promote better patient recovery than burr-hole drainage alone though further studies with larger sample sizes are needed for confirmation.

Another noteworthy finding was that the hemoglobin count was lower in the endoscopic group compared to the burr-hole group, and this difference remained statistically significant after multiple regression analysis, indicating a need for further investigation with an expanded sample size. Based on our intraoperative observations, the lower hemoglobin level in the endoscopic group is likely attributable to the larger incision required and the use of a milling cutter to create a small bone window, rather than significant blood loss during hematoma evacuation and lysis of fibrous membranes within the cavity. Furthermore, no cases of anemia were detected at the time of patient discharge, suggesting that the additional blood loss associated with the endoscopic procedure likely has little clinical implications. We also compared the impact of the two surgical approaches on electrolytes, liver function, kidney function, blood pressure, body temperature, and other parameters; most of these results showed no statistically significant differences between the two groups. The exceptions were sodium and alanine aminotransferase, which showed significant differences after multiple regression analysis, whereas potassium, chloride, and aspartate aminotransferase showed no significant differences. The results indicate that endoscopic-assisted evacuation does not cause dysfunction in other organs such as the liver, kidneys, or heart.

Previous multicenter clinical data have indicated that the hematoma recurrence rate in the endoscopic group is significantly lower than that in the burr-hole group (30), attributable to the endoscopic approach enabling direct visualization for evacuating the hematoma and lysis of fibrous membrane septations while performing electrocautery for hemostasis (14). However, the recurrence rate in the current study did not differ significantly between the two groups. This may be attributed to the limited sample size in the endoscopic group, as well as the exclusion of certain patients who underwent burr-hole drainage due to differences in anesthesia protocols. Furthermore, patients in the endoscopic group presented with significantly higher D-dimer values at admission, a factor that has been associated with increased recurrence rates (31). Notably, in a retrospective study by Yan et al. (32), the endoscopic group did not decrease the recurrence rate compared to the burr-hole craniotomy, consistent with the findings of the current study. Ultimately, randomized controlled studies directly comparing endoscopic-assisted hematoma evacuation combined with MMAE to other surgical modalities could provide robust evidence for further refinement of CSDH management guidelines.

## 5 Limitations

Although we collected as much data as possible from all patients preoperatively, postoperatively, and during follow-up, some data was inevitably missed. The small number of patients who underwent endoscopic-assisted evacuation also impacted on our final analysis outcomes. This retrospective analysis was also based on data from a single center, limiting the results' generalizability. Since endoscopic surgery requires training and skills that not all neurosurgical staff may have, inter-operator variations introduce a level of bias into the data. Additionally, current endoscopes and bipolar cautery devices are straight and non-flexible, which can lead to residual hematoma in deeper locations within the hematoma cavity and the inadequate clearance of some fibrous septa, thereby posing a potential risk for hematoma recurrence.

## 6 Conclusions

This retrospective analysis indicates that for CSDH, endoscope-assisted hematoma evacuation, leveraging clear and direct visualization, enables more precise and controlled hematoma removal, is associated with improved hematoma clearance and better functional outcomes without increasing complication rates, though it requires longer operative time and higher cost. These findings may help inform surgical decision-making and resource allocation for patients with varying clinical profiles.

## Data Availability

The raw data supporting the conclusions of this article will be made available by the authors, without undue reservation.
