# Supplementary material for: Endoscopic-Assisted evacuation vs. burr-hole drainage for chronic subdural hematoma: a retrospective comparative study
Source: Front Surg. 2026 May 12;13:1759497. doi: 10.3389/fsurg.2026.1759497 (PMC13201381; doi:10.3389/fsurg.2026.1759497)
Supplement: Supplementary file 1 [file Table1.docx]

**Table S1: Preoperative Vital Signs and Laboratory Measurements**

| Characteristics | Burr-Hole Group  N = 158 | Endoscopic Group  N = 40 | Total  N =198 | P-Values |
| --- | --- | --- | --- | --- |
| Temperature (℃), Mean ± SD | 36.52±0.25 | 36.50±0.18 | 36.52±0.24 | 0.4137 |
| Systolic Blood Pressure(mmHg), Mean ± SD | 132.90±17.52 | 133.80±15.38 | 133.08±17.07 | 0.7806 |
| Diastolic Blood Pressure(mmHg), Mean ± SD | 78.76±10.81 | 78.10±11.25 | 78.63±10.88 | 0.7321 |
| Mean Arterial Pressure(mmHg), Mean ± SD | 96.81±11.87 | 96.65±12.11 | 96.78±11.89 | 0.9392 |
|  |  |  |  |  |
| White Blood Cells(10^9/L), Mean ± SD | 6.53±1.99 | 6.50±1.85 | 6.52±1.95 | 0.9362 |
| Neutrophils(10^9/L), Mean ± SD | 4.38±1.76 | 4.51±1.82 | 4.41±1.77 | 0.6942 |
| Monocytes(10^9/L), Median (Q1-Q3) | 0.4 (0.3 – 0.6) | 0.4 (0.3 – 0.5) | 0.4 (0.3-0.6) | 0.6408 |
| Lymphocytes(10^9/L), Mean ± SD | 1.46±0.56 | 1.48±0.50 | 1.46±0.55 | 0.8614 |
| Platelets(10^9/L), Mean ± SD | 218.80±80.23 | 211.70±68.34 | 217.36±77.84 | 0.6042 |
| Hemoglobin(g/L), Mean ± SD | 136.90±14.64 | 137.10±14.52 | 136.96±14.58 | 0.9456 |
|  |  |  |  |  |
| Potassium（mmol/L), Mean ± SD | 3.92±0.35 | 3.88±0.28 | 3.91±0.34 | 0.5131 |
| Sodium（mmol/L), Mean ± SD | 138.70±3.27 | 138.60±3.52 | 138.69±3.30 | 0.8178 |
| Chloride（mmol/L), Mean ± SD | 104.70±3.40 | 104.10±3.78 | 104.59±3.48 | 0.3693 |
|  |  |  |  |  |
| Albumin(g/L), Mean ± SD | 40.00±4.46 | 39.82±4.09 | 39.96±4.37 | 0.8281 |
| Aspartate Aminotransferase(IU/L), Median (Q1-Q3) | 20.5 (17.3 – 26.2) | 20.2 (16.8 – 25.5) | 20.5 (17.2 – 25.8) | 0.2908 |
| Alanine Aminotransferase(IU/L), Median (Q1-Q3) | 16.6 (12.3 – 22.2) | 17.0 (12.9 – 24.2) | 16.7 (12.4 – 22.2) | 0.6551 |
|  |  |  |  |  |
| Creatinine（umol/L), Mean ± SD | 69.14±22.56 | 68.92±13.82 | 69.09±21.01 | 0.9536 |
| Blood Urea Nitrogen（ng/mL), Mean ± SD | 5.65±2.41 | 5.83±1.85 | 5.69±2.30 | 0.6033 |
