# Supplementary material for: Endoscopic-Assisted evacuation vs. burr-hole drainage for chronic subdural hematoma: a retrospective comparative study
Source: Front Surg. 2026 May 12;13:1759497. doi: 10.3389/fsurg.2026.1759497 (PMC13201381; doi:10.3389/fsurg.2026.1759497)
Supplement: Supplementary file 2 [file Table2.docx]

**Table S2: Postoperative Vital Signs and Laboratory Measurements**

| Characteristics | Burr-Hole Group  (N=158) | Endoscopic Group  (N=40) | Total  (N=198) | P-Values |
| --- | --- | --- | --- | --- |
| Temperature (℃), Median (Q1-Q3) | 36.6 (36.3 – 36.8) | 36.7 (36.5 -36.9) | 36.6 (36.3 – 36.8) | 0.1476 |
| Systolic Blood Pressure(mmHg), Mean ± SD | 120.60±14.14 | 122.50±13.35 | 120.99±13.97 | 0.4377 |
| Diastolic Blood Pressure(mmHg), Mean ± SD | 68.11±9.61 | 67.93±1.56 | 68.00±9.64 | 0.9148 |
| Mean Arterial Pressure(mmHg), Mean ± SD | 85.61±10.03 | 86.13±9.81 | 85.71±9.96 | 0.7691 |
|  |  |  |  |  |
| Potassium（mmol/L), Mean ± SD | 3.93±0.38 | 3.89±0.32 | 3.92±0.37 | 0.6060 |
| Sodium（mmol/L), Mean ± SD | 137.60±2.91 | 138.00±3.04 | 137.71±2.93 | 0.4532 |
| Chloride（mmol/L), Mean ± SD | 103.10±3.42 | 102.90±3.50 | 103.05±3.43 | 0.8051 |
|  |  |  |  |  |
| Albumin(g/L), Mean ± SD | 36.62±3.94 | 36.43±3.95 | 36.58±3.93 | 0.7958 |
| Aspartate Aminotransferase(IU/L), Median (Q1-Q3) | 18.7 (15.5 – 23.1) | 17.4 (15.6 – 21.7) | 18.4 (15.6 – 22.9) | 0.5930 |
| Alanine Aminotransferase(IU/L), Median (Q1-Q3) | 15.0 (11.1 – 21.6) | 14.2 (10.7 – 21.0) | 14.9 (10.8 – 21.2) | 0.8013 |
|  |  |  |  |  |
| Creatinine（umol/L), Mean ± SD | 65.73±23.60 | 66.47±16.99 | 65.88±22.36 | 0.8254 |
| Blood Urea Nitrogen(mmol/L), Mean ± SD | 5.31±1.88 | 5.49±1.55 | 5.34±1.82 | 0.5915 |
